# National Shortage of Purified-Protein Derivative Tuberculin Products

**Published:** 2013-04-26

**Authors:** John Jereb, Sundari Mase, Terence Chorba, Kenneth Castro

**Affiliations:** Div of Tuberculosis Elimination, National Center for HIV, Hepatitis, STDs, and Tuberculosis Prevention, CDC

Tubersol, a product of Sanofi Pasteur Limited, is in short supply nationwide until at least the end of May 2013. Tubersol is one of two purified-protein derivative (PPD) tuberculin products licensed by the Food and Drug Administration (FDA). The manufacturer has notified CDC that 50-dose vials of Tubersol will remain unavailable until the end of May 2013 and that supplies of 10-dose vials are still being reestablished: the product is available only by contacting Sanofi directly (at https://www.vaccineshoppe.com/index.cfm? or telephone, 800-822-2463). JHP Pharmaceuticals, LLC, the manufacturer of Aplisol, the other PPD tuberculin product licensed by FDA, has notified FDA that the product is available in restricted quantity. Acute local shortages of Aplisol also have been reported to CDC by TB control officials, as health-care providers switch from Tubersol to Aplisol. The shortages of Aplisol probably will diminish as Tubersol supplies are restored to their preshortage availability in the normal distribution networks. This report advises public health officials, clinicians, and workers in occupational health and infection control about how to adapt to the shortage.

Two kinds of immunologic methods are used for detecting *Mycobacterium tuberculosis* infection: tuberculin skin tests (TSTs) and interferon-γ release assay (IGRA) blood tests. The indications for use of these tests are the same, although one or the other method is preferred for certain populations (1), and this could play a role in setting priorities when one of the methods is unavailable. Together, these tests are the only means for detecting latent *M. tuberculosis* infection, and they contribute to diagnosing tuberculosis (TB) disease. When findings such as chest radiography, nucleic acid amplification test of sputum, and/or mycobacterial cultures are sufficient for confirming or excluding TB, the results from a TST or an IGRA blood test might not be needed (2). A negative TST or IGRA result does not exclude the possibility of TB infection because some persons can have a compromised ability to react to tests for TB infection. However, most persons diagnosed with TB in the United States have had a positive TST or IGRA blood test that has contributed to that diagnosis. When TB disease is strongly suspected, specific treatment should be started regardless of results of a TST or an IGRA blood test (1,3).

In controlled studies, the agreement between TST results from Tubersol and Aplisol is high. The agreement between results from a TST and an IGRA blood test or between results from the two commercial IGRA blood tests is lower (1).

CDC recommends any of the following three general approaches for addressing the shortages of TST antigens:

1. Substitute IGRA blood tests for TSTs. Although the costs associated with the blood tests themselves can be greater than the cost of TSTs, the use of the blood tests might be more cost-effective in certain settings because their improved specificity in persons who have had previous Bacille Calmette-Guérin (BCG) immunization or exposure to nontuberculous mycobacteria might allow for better targeting of preventive therapy. The blood tests require phlebotomy, preparation of blood specimens, and specific laboratory services for analysis; these tests are not available in all practice settings. Clinicians who use the IGRA blood tests should be aware that the criteria for test interpretation are different from criteria for interpreting TSTs (1).
2. Allocate TSTs to priority indications, such as TB contact investigations, as determined by public health authorities. This might require deferment of testing some persons. CDC does not recommend testing persons who are not at risk for TB (4).
3. Substitute Aplisol for Tubersol for skin testing. In cross-sectional studies, the two products give similar results for most patients. Shortages of Aplisol are expected to become more widespread, limiting the feasibility of this approach.

Some surveillance programs for TB infection control rely on routine serial TSTs. Switching products or methods might make changes in serial results difficult to interpret: the apparent conversions of results from negative to positive or reversions from positive to negative could be caused by inherent interproduct or intermethod discordance (1,5). In settings with a low likelihood of TB exposure, the deferment of routine serial testing should be considered in consultation with public health and occupational health authorities.
